# Jarvis’s Adjuster for the Reduction of Dislocations, and Treatment of Fractures

**Published:** 1850-10

**Authors:** Theodore S. Bell

**Affiliations:** Louisville, Ky.


ART. III.—*Jarvis's Adjuster for the Reduction of Dislocations, and Treatment of Fractures.* By THEODORE S. BELL, M. D., of Louisville, Ky.

In a brief editorial notice, in the last number of this Journal, reference was made to an admirable apparatus, entitled Jarvis's Adjuster, and its merits are such, in my estimation, as to entitle it to the confidence of the medical profession. In order to present its claims fairly, and to advance the usefulness and certainty of surgery, I undertake a description of the instrument, and of its powers. As a preliminary to this, however, it will not be amiss to notice the subject of dislocations and fractures.

In the preliminary remarks of Sir Astley Cooper, to his invaluable work on Dislocations and Fractures, that distinguished writer endeavors to impress upon the minds of surgeons the exceeding importance of a correct understanding of the pathology of dislocations. There are many deplorable examples of mistakes, blunders, and failures, in almost all sections of the world, growing out of this very ignorance, and an unreduced dislocation is a monument, to a surgeon, as durable, as far as his surgical life is concerned, as brass or marble. No man should attempt the reduction of a dislocation, unless he has a thorough and minute understanding of everything that he may have to encounter in the reduction. If an extending force is improperly applied, an immense amount of unnecessary power may be exerted, where little is required, if it is correctly, judiciously, and skilfully directed. Columbot undertook, some years since, to show that no mechanical aid, and no assistants of any kind were of use in the reduction of any kind of dislocations. He made the patient exert the counter-extending power, while the surgeon skilfully rotated the head of the bone to its proper place. Many respectable surgeons then imagined that the ambe of the ancients, and the pullies, and the stout assistants of the moderns were about to be consigned to oblivion. But a little experience caused the illusion to vanish—the pullies have maintained their place, and deserve some reputation.

The importance of a thorough understanding of the pathology of the shoulder joint dislocation is so great, that no apology is necessary for introducing here the excellent remarks of Mr. Crampton on the subject, which were published in the seventh and eighth numbers of the Dublin Journal. Sir Astley Cooper gives, in his work on Dislocations, but two dissections of luxated shoulders, and both of those were dislocations into the axilla. Mr. Crampton's investigations shed a good deal of light, however, on other kinds of dislocation of the shoulder, and the pathological appearances in the same species of dislocation, as that described by Sir Astley, vary somewhat from Sir Astley's description. It is well enough for the surgeon to remember a portion of Sir Astley's description. He found that "the resistance to reduction (even after death,) was such as he could not by himself overcome; he divided one muscle after another, cutting through the coraco-brachialis, teres major and minor, supra-spinatus muscles, but still the opposition to his efforts remained; he next divided the deltoid muscle, and found that the supra-spinatus muscle was his great opponent, *until he drew the arm directly* upwards, when the head of the bone glided into the glenoid cavity."

A surgeon who has had much to do with dislocations of the shoulder, cannot fail to place the proper estimate upon such facts as these, and by way of impressing the subject upon the attention of the reader, the investigations of Mr. Crampton are added.

Mr. Crampton dissected a dislocation of the shoulder, caused by an injury which produced the death of the sufferer in two hours after he met with the accident. "When the axilla was cleared, the head of the os humeri was seen lodged on the inferior costa of the scapula, or rather on its neck; the head of the bone, in escaping from its socket, had pushed the teres minor downwards, and burst through the lower part of the subscapularis muscle, some of the fibres of which closely embraced the neck of the bone, while the bulk of the muscle was pushed upwards and detached from the inner surface of the scapula. The neck of the humerus, therefore, was in some degree embraced by the divided fibres of the subscapularis muscle, while a portion of its head rested on the neck, and part of the venter of the scapula, without the intervention of any muscular substance. The short head of the biceps and the coraco-brachialis were forced to describe a curve outwards over the neck of the humerus on the sternal side, while the long head of the triceps crossed the neck of the bone obliquely on the dorsal side; this strangulation of the head of the bone, by the surrounding muscles, was made most apparent when extension was applied to the fore-arm. The biceps and triceps seemed then to close behind the head of the bone, and interpose themselves between it and the glenoid cavity; the tendon of the long head of the biceps remained in its groove, but the sheath in which it runs was partially ripped up.

"The capsular ligament was completely torn from the lower part of the neck of the humerous to the extent of more than half its circumference, the torn edge appearing like a crest over the head of the bone. * * * The tendons of the supra-spinatus, infra-spinatus, and teres minor were completely torn from the humerus, carrying with them a scale of bone from the tubercle.

"To ascertain the obstacles opposed to reduction, the scapula was fixed, the arm raised, and extension applied to the wrist. So long as the hand was held *supine*, the head of the bone remained immoveable, apparently from the closing of the biceps and triceps behind it. On turning the hand *prone*, rotating the limb inwards, and continuing the extension, reduction was easily effected.

"The appearances in this case differed from those observed by Sir Astley Cooper, and agreed with those related by Mr. Thompson. In the case of the latter gentleman, the head of the bone was lodged on the inside of the neck of the scapula, between the subscapularis and teres major—the capsular ligament was completely torn from the whole circumference of the humerus—the attachments of the tendons of the supra-spinatus and infra-spinatus were torn off, with the part of the bone they were inserted into—and some fibres of the subscapularis encircled the neck of the bone. In Sir Astley Cooper's case, on the contrary, the tendon of the subscapularis was torn through, but the supra-spinatus and infra-spinatus remained attached to the tubercle, and reduction could only be effected by relaxing these muscles. Mr. Crampton observes very justly that the comparison of these facts is sufficient to shew, that in apparently similar dislocations of the humerus there may be very different degrees of lesion, and consequently different causes of resistance to reduction.

"J. W. æt. 30, was precipitated twice consecutively into a burning lime-kiln, from a height of about fifteen feet. He was carried to the Meath Hospital. In addition to severe burns and lacerations, there was a dislocation of the humerus under the pectoral muscle, which Mr. M'Namara himself reduced, by merely drawing the arm gently forwards and downwards with one hand, while he pushed the head of the bone towards the glenoid cavity with the other. The man died in the course of the day, and eighteen hours after death the joint was dissected.

"The dislocation was unattended with the rupture of any muscle, or the separation of any tendon from its insertion into the bone: by a slight effort the dislocation was reproduced, and the pectoral muscles being removed, the polished head of the bone was now seen lodged on the cervix of the scapula, at the root of the coracoid process, but extending nearly as far as the notch in the superior costa; it had passed out through a rent in the capsular ligament, *over* the *upper edge* of the tendon of the subscapularis, detaching this muscle from its connexion (which at this point is but slight) with the inner face of the scapula, and pushing its fibres downwards, so that they formed a curve which partly embraced the neck of the humerus; the supra and infra-spinatus muscles were on the stretch, but had suffered no injury. The cellular substance covering their tendons was deeply ecchymosed, so as to mark their course most distinctly. On replacing the head of the bone, the opening in the capsular ligament, through which it had escaped from its socket, could be distinctly seen. It was formed by a separation of the ligament from the interior side of the brim of the glenoid cavity from top to bottom; it was bounded at the top by the tendon of the supra-spinatus, and at the bottom by the inferior edge of the tendon of the subscapularis, the rent was continued as far as the root of the lesser tubercle of the os humeri, and was of sufficient extent, but no more, to permit the head of the bone to pass easily through it; the *inferior part of the capsular ligament*, *however*, (*the part corresponding with the axilla*,) *was perfect*.

"The great blood vessels and nerves lay to the sternal side of the head of the humerus, and were forced a little out of their course. The axis of the head of the bone, in its dislocated position, was scarcely a quarter of an inch higher than the axis of the glenoid cavity.

"Mr. Crampton appends to these interesting and valuable facts some practical observations. We will endeavor to give their substance.

"I. Resistance to the reduction of a recently dislocated shoulder would seem to be owing to spasmodic contraction of the muscles, and *not* 'from the neck of the bone being tightly embraced by the ruptured capsular ligament.' Dissection does not prove the latter, and therefore it is mere supposition. Muscular contraction then being the obstacle, we must diminish its power by bleeding, the warm bath, antimony, long-continued extension. Another excellent means of obviating muscular oposition is taking the muscles by *surprise*.

"If before assistants are called in, or any apparatus is applied, the surgeon, while he appears to be occupied merely in ascertaining the nature of the injury, applies a gentle extension at the wrist, and slowly raising the arm to nearly a horizontal position, suddenly pulls it upwards and a little forwards, (that is towards the patient's face,) while at the same time he as suddenly pushes the trunk backwards by pressing, with the left hand, below the axilla, he will in a great number of *recent cases* succeed by this simple process in reducing the dislocation. His success, however, will in a great measure depend on the *unexpectedness* of the attempt; he should, therefore, endeavor to divert the patient's attention from his proceedings, and I know of no means so effectual for this purpose, as inducing him to describe circumstantially everything connected with the occurrence of the accident; this is a theme on which all patients, who are at all able to express themselves, are sure to expatiate with the greatest satisfaction, and once engaged on so engrossing a topic, it will require but a small degree of tact on the part of the surgeon to seize the favorable moment when he can apply his force with the greatest advantage.'

"II. In luxation into the axilla, muscular contraction seems to operate chiefly by pressing the head of the humerus against the inferior part of the brim of the glenoid cavity; in Sir Astley Cooper's case this was mainly effected by the supra-spinatus. The obvious deduction is, to raise the arm to nearly a right angle with the body previous to extension, and not to use any force in pressing the head of the humerus upwards, as that must lock it more firmly.

"III. The *direction* of the extension is now generally that of a right angle with the body. In the reduction by the heel in the axilla the direction is nearly parallel with the body, but Mr. C. thinks that more force is necessary with this method. Mr. White, of Manchester, drew the limb directly upwards, but the practice has fallen into disuse—a presumptive proof of its not possessing any superior efficacy. The method has lately been brought forward at the Hôtel Dieu as a novelty. We are not surprised at this. M. Dupuytren has no love for English surgery, and candor forms no part of his merits or acquirements.

"IV. Extension is now pretty generally applied to the wrist in preference to the arm. It is less painful, if not more effectual.

" 'V. Great stress is laid by most surgeons on the advantage of fixing the scapula, as it is called; it may be doubted, however, whether the thing be possible, or if possible, advantageous. It is quite plain that a split cloth, or a napkin with a hole through which the arm is passed, can, when the arm is strongly extended, acts only on the inferior costa of the scapula, or rather on the walls of the axilla formed by the edges of the latissimus dorsi, teres major, and pectoralis major muscles; the whole effect of this force can be no other than to push the inferior angle of the scapula backwards and upwards, consequently to direct its superior angle, and the glenoid cavity downwards, and, by acting on the pectoralis major and latissimus dorsi, to draw the head of the humerus inwards towards the ribs, that is, to remove it *from* the glenoid cavity. To obviate this objection, some surgeons recommend pressure to be made by the hand of an assistant on the acromion of the scapula, so as to push it backwards while the humerus is drawn downwards and outwards; but it is plain that unless the force which the surgeon applies to the head of the scapula to keep it back, be at least equal to the extending force which is applied to the arm, the scapula cannot be *fixed*, it *must* follow the arm; besides when the arm is raised, the deltoid fills up the sub-acromial space and renders it impossible to apply any appreciable force to the acromion. As the neck of the scapula cannot be pushed *upwards*, it is proposed by Bonn, to disengage the bones by pressing the head of the humerus *downwards*, at the moment when the extension is at the utmost; the proposal is a most rational one, and has been adopted for several years past in the County of Dublin Infirmary, as I think, with considerable advantage.'*

"VI. When a considerable power of extension is required, Mr. Crampton prefers a ladder to the pullies. One end of the ladder is fixed—the patient stands between the bars near this end—the wrist of the affected limb is tied by a jack-towel or handkerchief to the sides of the free end of the ladder at a convenient distance from the body—and thus a powerful lever is obtained,

*Contrast this with Mr. Toogood's brief remarks on fixing the scapula.

one end of the ladder fixed on the ground being the fulcrum, the other end being free, and its depression, by an assistant producing the extension. Counter-extension is, of course, effected by a towel under or in the axilla.

"VII. It has been much mooted whether dislocation forwards is a *primitive* dislocation, and it has been supposed that every luxation is primarily into the axilla. The case related by Mr. Crampton seems to prove that it is, or at least in that instance it was a primitive dislocation, and as the inferior part of the capsule was not ruptured, it would of course have been injurious to have first reduced the head into the axilla. In such a case the clear indication is to force the head of the bone *backwards* towards the glenoid cavity, the axis of which is as nearly as possible in a line with that of the head of the humerus; this can be effectually done by applying a fulcrum immediately below the axilla, and using the dislocated arm as a lever of the first kind; the surgeon should therefore place his left arm extended horizontally, immediately below the walls of the axilla, between the dislocated arm and the chest, and then grasping the wrist in his right hand, he should draw the arm forcibly across the patient's body."

These remarks are copied from the Medico-Chirurgical Review for 1833, and the practical bearings of the pathological facts must fasten themselves upon the mind of the surgeon. There are many dislocations of the shoulder that are manageable almost without any mechanical contrivances, but there are others that resist all the ordinary methods, and it is in cases of this kind that Mr. Crampton's pathological facts rise to importance. Malgaigne has described a luxation of the humerus, which has been considered an impossible form for that accident. The head of the humerus was carried forward and upwards, above the coraco-acromial ligament and coracoid process. The projection was so great that it extended for two inches beyond the anterior surface of the clavicle; and it was so superficial that, at some parts, it seemed subcutaneous." Malgaigne's opinion was, "that the head of the bone had at this spot separated the deltoid and great pectoral, so as to become almost subcutaneous." This is certainly a very remarkable case of dislocation, and shows the necessity of fullness of knowledge upon all possible varieties of dislocation of the shoulder, and upon all the obstructions that interfere with reduction.

Professor Rozer, of Tubingen, describes a variety, discovered in a dissection made by himself, in which the head of the humerus laid in front of the short head of the biceps, the sub-scapular muscle was ruptured, and completely detached from the lesser tuberosity of the humerus, and the head of the bone elevated the scapular extremity of the pectoralis minor. This dislocation had taken place seven years previous to the death of the subject, and remained unreduced. The obstruction to the reduction was the interposition of the short head of the biceps between the head of the humerus and the glenoid cavity. It could have been reduced by proper rotation.

I do not purpose to enter into a disquisition at this time, upon the many means in the way of mechanical contrivances, that have been proposed for reducing dislocations of various kinds. The great desideratum in these matters is that which will most effectually overcome the resistance of the muscles up to a certain point, which gives the surgeon the utmost freedom of motion in the luxated limb, and which enables him, at the proper moment, to cease the extension of the muscles, and instantly to command their services in the reduction of the luxation. Mr. Spong, Mr. Skey, and Dr. Gilbert have proposed various measures for these objects, but their methods looked to extension power, and neglected by far the most important object. They antagonised the muscles admirably, but could not then seize upon their aid at the moment it should have been given, nor could they enjoy the proper amount of freedom in moving the limb, for the purpose of clearing away obstructions. The pullies are open to the same objections. Mr. Epps, of University College, London, in 1845, made a partial approach to the invention of Dr. Jarvis, but his apparatus was defective in so many particulars, that it made no impression upon the surgeons of the day.

The profession has, in the pullies, an apparatus of sufficient power for extension, and counter-extension. If those objects were all that are involved in the reduction of dislocations, the pullies would leave nothing to be desired. But in every form of luxation there are more important objects than extension and counter extension; in some dislocations of the hip, the extending qualities of the pullies are to a considerable extent, a disadvantage, because of their interference with other necessary measures. Mr. Vincent, an accomplished surgeon, seems to have laid down the principle of action, that should guide in nearly all dislocations. He says: "The surgeon cannot be said to effect the reduction. His efforts are limited to drawing the bone so near the glenoid cavity as to bring the muscles into a situation to remove that association in which all these actions have been accustomed to take place; in other words, in which they all concur to draw the bone into its normal position and retain it there." The true principle is to command the contractile power of the muscles, so far as it is exercised upon the abnormal position of the bone, and at the moment the bone is ready to resume its natural place, the aid that overcame the resistance of the muscles should be cut loose, to use a figurative expression, and the muscles will then perform their duty.

The following remarks, copied from the London Lancet, January 3, 1846, present the subject in a very forcible point of view. The Lancet says: "Dr. Jarvis remarks that the first object in the reduction of dislocations is to overcome the resistance offered by the muscles, by their power of approximating their points of attachment, for which extension and counter-extension are necessarily employed, and either manual assistance or the pulleys are resorted to, both of which act mechanically alike in producing traction on the limb; attention is then drawn to the fact that they both act from fixed points over which the surgeon can have no control, so that if the line of extension happen to be correct he does not desire it changed, but if it happen to be incorrect the force must first be relaxed before he can change it.

"Thus in luxation of the femur into the ischiatic notch, in consequence of the oblique position which the head and neck occupy to the shaft of the bone, any force applied to the head from the other end of the shaft and in a line with it, necessarily forces the head in some degree (while passing through the muscle) to follow the obliquity of the neck, and not the line of the shaft, thus making an oblique track from the cotyloid cavity. Suppose pulleys applied, the head and neck being forced obliquely into the muscular tissue, as not unfrequently happens, we have power constantly and steadily operating from fixed points, and before the direction of that force can be changed we must relax it. The head and neck, from their oblique position to the shaft, especially since the head is larger than the neck, would be likely to operate like a hook around a portion of muscle which lies directly below them, and before they could be disengaged, the force operating on one line would be likely to tear through the muscle or break the bone—a case which has happened more than once. But if, instead of the extending point being fixed, as in the case of the pulleys, it is left free, and subject to the command of the surgeon, while the force is steadily and constantly applied, the surgeon would be much more likely to disengage the head by giving free motion to the limb, than he would by applying that force in one given line. Here is another error in applying mechanical principles to surgery.

"In some dislocations of the os humeri, where the head of the bone is driven into the soft parts, and the neck is closely embraced by them, we must first disengage the head before we can hope to succeed in reduction. With the pulleys, force is steadily applied, but owing to the firmness with which the neck is embraced, and the strength with which the muscles contract, the bone is not relieved from its confinement. If the extending point was subject to the will of the surgeon, to move at pleasure, while the force was yet constant and steady, the giving free motion to the limb would greatly aid him in reduction, and that too without increasing the power.

"In the four dislocations of the femur, Dr. Jarvis' instrument may be applied so as to have any amount of power available which we require, and in whatever direction we need, and at the same time to leave the limb just as free for motion as it was without the instrument attached to it. All the indications required are fulfilled without the extending point being fixed, as by the pulleys or with assistants. In a dislocation of the bone upwards and outwards, the head resting on the dorsum ilii, if you wish to move the limb to disengage the head of the bone from the gluteus minimus, into which it has been driven, while all the power which you have applied is retained, you can do so. The limb can be moved with the same facility as before the instrument was applied. The head of the bone can be thrown from the dorsum ilii while an assistant rotates the thigh on its own axis, a side lever being at the command of the operator which does not in the least affect the line of extension.

"In a dislocation into the ischiatic notch the side lever is used merely to lift the head of the bone from the sacro-sciatic notch, while the extending force is applied to bring it forwards from that deep situation to the acetabulum.

"In a dislocation on the pubes the head of the bone is out of the line of the upper third of the ilium and the inside of the knee, some two or three inches; it lies above a transverse line passing through both acetabula about an inch. The greatest amount of force to reduce this dislocation is generally required to operate transversely to the body, yet much force also is required to restore the normal length of the limb. With this instrument properly adjusted, while the extending force is applied, the instrument and limb may be gently but firmly carried outwards. The instrument itself becomes a lever, a strap crossing the dorsum of the ilium the fulcrum and the head of the bone the body to be moved, and the surgeon gently applies his own force to the end of the lever as the moving power.

"By an entire change in the application of the power the instrument is made available in luxations into the foramen ovale."

"In the 'Gazette des Hôpitaux,' Dr. Huet states that "in Dr. Jarvis's instrument we have a means of extension and counter-extension, powerful and at the same time regular, subject to the will of the operator, and easy of application. There will no longer be occasion to look around for fixed points of extension and counter-extension, these points departing directly from the centre of motion of the instrument. The operator will have at his disposal an enormous power which the most vigorous muscles cannot resist. He may increase it, diminish it, apply it rapidly or slowly, according to the resistance of the muscles, without danger of injuring them in any manner; and he can act with facility upon the mobility of the dislocated limb during the whole period of reduction. These and many other advantages are enumerated; we have examined the instrument, the cost of which is ten guineas; we can testify to its ingenuity, and it appears to us to be correct in principle, and capable of fulfilling the purposes professed; but practical experience alone can determine definitively as to its real utility."

In the reduction of old luxations, these principles are, if possible, more valuable than in recent cases. In every possible point of view, the adjuster of Dr. Jarvis is infinitely the best apparatus I have seen for reducing every species of dislocation that can be aided by any mechanical contrivance, whether it is a luxation of the hip, shoulder, thumb, or fore-arm. The adjuster is available at any moment its services may be needed. As Dr. Huet says, it is unnecessary to hunt up fixed points, as we have to do for pullies. It can be used in an open field, at the road side, or at any other place where accident may require its use. While it gives the surgeon an apparatus for extension and counter-extension, superior to all others, it enables him to move the dislocated limb with great freedom, and to use the muscles, by an instantaneous stoppage of the extension power, for the completion of the reduction. These are the eminent advantages of Jarvis's adjuster, and well entitle it to the confidence of surgeons. If it is properly used, it cannot fail to grow upon the esteem of those who desire its services.

Before dismissing the subject of dislocations, it may not be improper to refer to the experience of the profession on the age of dislocations that may be reduced. Surgical authorities are somewhat at variance with surgical experience on this point. The usual admonition is not to attempt a reduction if the bone has been out of its place more than a month; and this too against the matured experience of Desault! Dieffenbach reported a case in 1840, in which he reduced a dislocation of two years' standing, of the shoulder. Instead of resorting to force, to break up adhesions, he introduced a small knife through the skin, while the arm was well extended, and divided the most tense portion of the pectoralis major; near its tendon. The knife was then introduced at the posterior part of the border of the axilla, and the latissimus dorsi, and teres major and minor were divided. *After this the knife was introduced at three different points near the humerus, for the division of the dense, hard, false ligaments which surrounded the artificial joint. By judicious rotation, the head of the bone was then restored to the glenoid cavity. Proper bandaging restored the limb, in a short space of time, to its usefulness.

Dieffenbach also reports a reduction of a dislocation, of a year's age, of the ancle joint. The reduction was effected by the section of the tendo-Achilles. The cure is reported to have been perfect.

M. Sedillot, surgeon at the Val de Grace, reports a case of luxatlon of the humerus backwards into the fossa infra-spinata, which was reduced one year and fifteen days after the dislocation occurred.

Dr. Stark, in the Edinburgh Medical and Surgical Journal, for January, 1848, reports a case of dislocation of the head of the radius, successfully reduced two years and one month after the occurrence of the luxation.

It is unnecessary to burthen this paper with many cases of the kind referred to. There is abundant reason even in these, though they stood alone, to encourage judicious, patient and persevering attempts at reducing old dislocations. Surgery should be a progressive science. I am satisfied that with Jarvis's adjuster, and a prudent, carefully guarded administration of pure chloroform, much more success in the reduction of dislocations, both recent and old, may be anticipated, than can be hoped for without those agents. Dr. Gregory has taught a sure method both of purifying chloroform, and of testing its purity, and statistics seem to establish the fact, that if pure chloroform is administered to a person in a horizontal position, and on an empty stomach, there is no danger to be apprehended from its use.

In addition to the eminent advantages of Jarvis's adjuster, in the managent of dislocations, it has many claims to confidence in the treatment of fractures, among those who are wedded to splints, and will not forsake their idols. The adjuster certainly fulfils all the indications, in the treatment of fractures, which the experience of surgeons has deemed essential, better than any splint that has ever been devised. In this Journal, for November, 1849, I quoted M. Bauden's rules of treatment, and demonstrated, I think, that all the apparatus in ordinary use failed to fulfil the objects of the surgeon. These very failures are the basis of most of the suits at law, for mal-practice, and a distinguished Professor has recently claimed Professor Hamilton's statistics of fracture as evidence that juries should not look too confidently to surgery, for success in the treatment of fractures! It is hoped that surgeons will rather look upon those statistics as a most excellent reason for examining into the causes of failure, and for the discovery of better means of treatment. To those who have faith in the use of splints, I would commend the adjuster, as the most reliable and certain aid they can find in mechanical apparatus in the management of fractures. This instrument maintains the most perfect extension and counter-extension, and secures the coaptation of the fractured ends of the bone. Unlike other instrumentation, which does generally quite as much evil as good, the adjuster performs its part without that mixture of evil, which prevails under all the modifications of Desault's splint, and of the inclined plane, as it is generally used.

There are those who object to the adjuster because the inventor protected his rights to his instrument, by the patent laws. I can see but a dim shadow of analogy between cases of this kind, and patent medicines. The latter are the production of an impudent cupidity, that is altogether reckless of the evil that may be done, provided the pockets of the charlatan are filled. His catholicon is not placed in the hands of those who know how to use it for the benefit of sufferers, but is sown broadcast over the land, to be swallowed by the ignorant and the credulous, for every variety of imaginary or real ailment. The patent right in Jarvis's adjuster has no resemblance to such things as these. It is in the nature of the copy right in a book, or an anatomical atlas, and no one imagines that a medical author does any wrong by seeking the protection of the laws of copy right. If one is professional the other is equally so. The inventor of a useful, ingenious, complicated set of instruments should guard the integrity of his instruments, by securing their manufacture in the best possible style, and he has as good reason for protecting them from piratical instrument makers, as the author has for guarding himself from piratical publishers. When a surgeon uses a patented instrument he knows what he is doing, but when a physician prescribes a secret nostrum he works in darkness, and exhibits a culpable recklessness.

I have merely given a view of the merits of Jarvis's adjuster, and mentioned the objects that can be attained by it. It would be impossible to convey a correct idea of its peculiar construction, without illustrative drawings, and I cannot describe it, beyond saying that the instruments acts upon the pinion and ratchet principle; this produces counter extension, through a fork for the hip, shoulder, or elbow, as the case may be, and the power of extension is perfect. Pads, straps, belts, &c., form a part of the instrument. An inclined plane for fractures is also a portion of the apparatus.

But though I cannot describe Jarvis's real contribution to the progress of surgery, those who may desire to understand the inventor's method of reducing dislocations, and of treating fractures may do so by addressing Dr. George O. Jarvis, Portland, Connecticut, or George

Kellogg, A. M., Birmingham, Derby, Connecticut. The lectures of Dr. Jarvis, published in the London Lancet, in 1846, are among the most sensible teachings in surgical literature, and will amply repay any surgeon or physician who may study them. While the author of those lectures was in Europe he won the favor and approbation of the enlightened professional men of France and England. Sir Benj. Brodie, Mr. Quain, editor of a valuable anatomical work, S. Cooper, author of "First Lines of Surgery," and "Dictionary of Practical Surgery," R. Keate, Surgeon to St. George's Hospital, W. Ferguson, author of "Practical Surgery," Thos. Hodgkin, lecturer on morbid anatomy of the serous and mucous membrane, E. Stanley, President of the Royal College of Surgeons, "The London Medical Gazette," "Gazette des Hopitaux," "Archives Generales de Medecine," and a number of prominent American surgeons, have borne strong testimony to the great merits of Dr. Jarvis's adjuster. I am satisfied that a medical man who makes himself familiar with Dr. Jarvis's lectures, and adjuster, will not willingly do without the instrument. He may secure success in cases, where there is frequent failure, and constant uncertainty without it.

